# Production and characterization of human hair keratin bioplastic films with novel plasticizers

**DOI:** 10.1038/s41598-023-44905-x

**Published:** 2024-01-12

**Authors:** Anand Shubha, Gupta Sharmita, Lakhani Anita

**Affiliations:** Dayalbagh Educational Institute, Dayalbagh, Agra, Uttar Pradesh 282005 India

**Keywords:** Biotechnology, Environmental sciences

## Abstract

Since their invention, conventional plastics have contributed in the betterment of the society in numerous ways, nevertheless their deleterious impacts on the natural ecosystems and living creatures is irrefutable. The management of plastic waste generated is a concern worldwide and therefore quest for the plastic alternates or bioplastics is imminent. Here, we explore the suitability of keratin from human hair waste as the candidate for the production of bioplastic films. Keratin extracted from hair was used to form the films or ‘kertics’ by solution casting and curing. Ethanediol, di-ethylene glycol and tri-ethylene glycol were used as novel plasticizers along with glycerol in the keratin film formation. The film prepared were of the thickness 190–220 µm with the area of about 4.54 ± 0.2 cm^2^. Water uptake by G100, ED100, DEG100 and TEG100 films was recorded to be 4.8, 6.2, 4.9 and 6.3% respectively. FESEM analysis revealed that the films with 100 µl of 1% glycerol (G100) had continuous surface morphology except few pits of 0.1 µm, also DEG100 and TEG100 films have the most uniform surface morphology with no evident pits, holes or bulges. X-ray diffractogram showed characteristic peak of keratin at 19.5° and the d-spacing value observed was 0.45 nm. The FTIR studies suggested that the films retained keratin in non degraded form, and possessed the characteristic Amide peaks. The films were also found to be biodegradable in studies involving keratinophilic fungal strain of *A. oryzae.* These films could found potential applications in packaging industry, disposable items manufacturing and biomaterial generation.

## Introduction

Since 1860, when the very first plastic was invented^1^, about 8.3 billion tonnes of plastic has been manufactured worldwide and 6.3 billion tonnes of it is waste^2,3^. As far as recycling of plastic waste is concerned only 8.7% of this waste has been recycled so far. Plastic imparts uncountable ecological as well as biological impacts^4^ on the living and non living components of our ecosystems^5,6^. Considering these deleterious effects of plastics newer biodegradable alternatives are sought and therefore varied biological waste or other renewable raw materials are being explored to produce plastic alternatives. Biodegradable polyesters, polylactic acid, polysaccharides and proteins from plants or animal sources are few of the materials being explored to produce plastic alternates^7–14^. Researchers around the globe are continuously thriving to improve the performance and properties of these bio-based plastics to maximize their applications in packaging and manufacturing of disposable items/ medical devices.

Various proteins from plant and animal sources have gained heightened interest in past few years for the production of bioplastics. The interactions of protein molecules because of the range of functional groups present in amino acid side chains give them additional benefit over other biomolecules. The protein bioplastics have the properties of safety, biocompatibility and biodegradability^15^. The isolated proteins from various sources have demonstrated the property of denaturing and again renaturing under laboratory condition which allow them to be plasticized and obtain films with suitable properties. Collagen, Milk Proteins (casein and whey), Myofibrillar proteins, keratin and gelatin includes the animal proteins being studied for the synthesis of bioplastics^16–20^. Keratin bioplastic films from sources such as chicken feather, duck feather and sheep wool and there characterization have also been reported^21–24^.

Human hair is made up of up to 80% of hard keratins. Amino acid Cystein (Cys) is present in large amount in hair keratins that gives them strength and stiffness as result of disulphide linkages^25,26^. For the extraction of human hair keratin different approaches could be applied including oxidation and reduction procedures^27^. The oxidized form of keratin is known as keratose and the reduced form is known as kerateine^28^. Extracted keratin from human hair has been reported to be used for the synthesis of films, fibers, sponges, Hydrogels and composites for various applications. Poly(ε‐caprolactone)—keratin fibers have been reported to possess structural integrity in the ratio 70:30^29^. Human hair keratin films with 1% glycerol as plasticizers have also been reported but the effect of varied concentration of glycerol have not been reported so far^30,31^. Application of ohmic heating has been reported to produce keratin films and it was found that the films formed with conventional heating had better surface morphology^32^.

In the present research keratin protein derived from waste human hair is being utilized to produce the bioplastic film. We would like to coin the term “kertics” for all the keratin based bioplastics. The present research aimed at studying the effect of different concentrations of glycerol as plasticizer on the properties of human hair keratin film. Also, ethanediol (ED), diethylene glycol (DEG), triethylene glycol (TEG) are being reported as the novel plasticizers for keratin film in this research for the first time. Method of solution casting in silicon moulds was adopted to obtain the final stable films after curing procedures. Silicon moulds were adopted in place of polyethylene plate to cast the films owing to flexibility and ease of film removal, non-sticky surface as well as reusability. Characterization of the bioplastic film produced was done by various analytical techniques including field emission scanning electron microscopy (FESEM), X-ray diffraction (XRD) and Fourier-transform infrared spectroscopy (FTIR). Biodegradability of the films was also studied by keratinophilic fungal isolate *A. oryzae* obtained from soil by hair baiting technique. The outcomes of the present study shall contribute to the understanding of effect of various plasticizers and their concentration on the film properties as well as their suitability for packaging and other applications.

## Results

**Figure 1:**
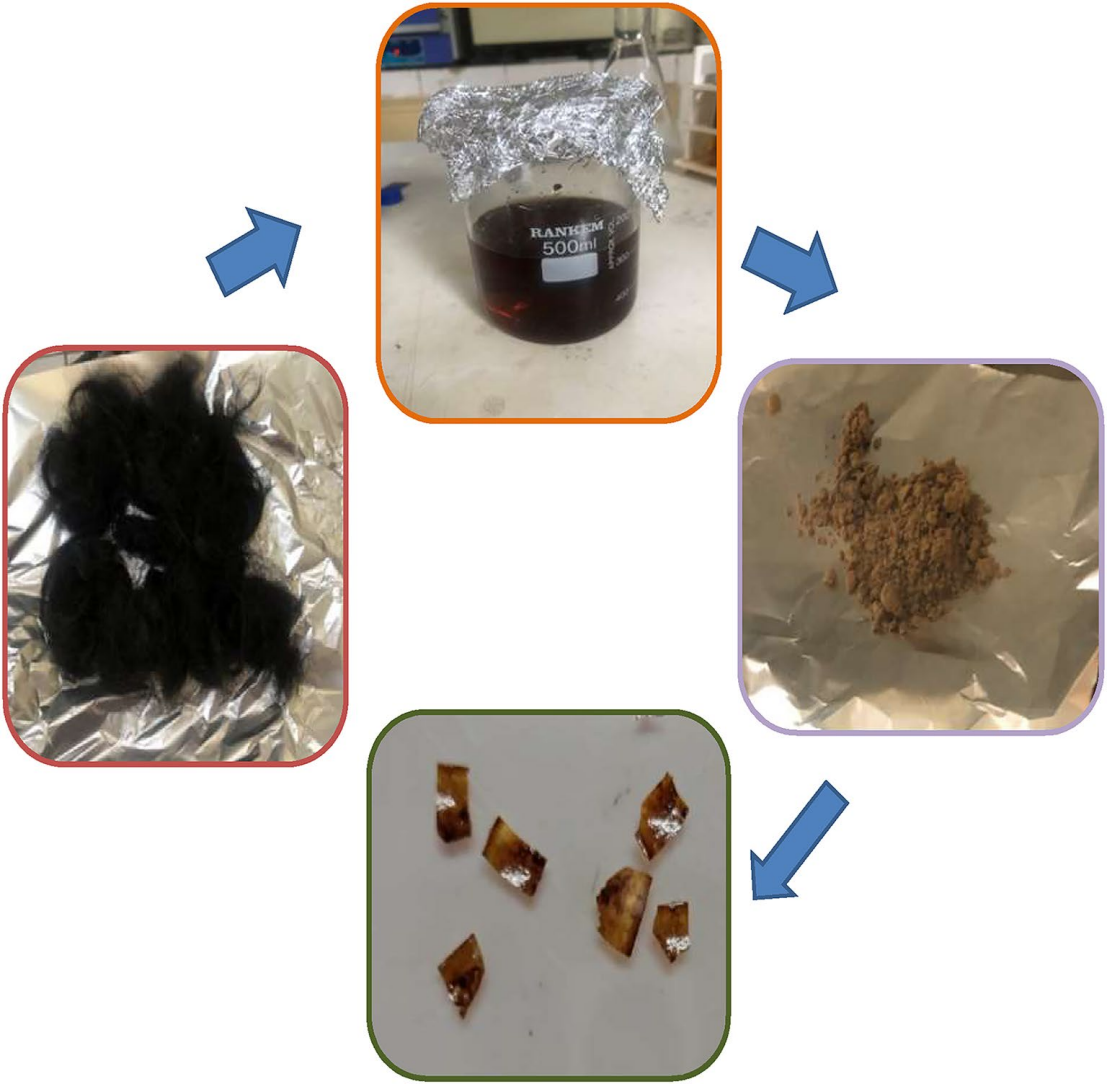
Overview of bioplastic production. Clean hair samples were delipidized in chloroform and methanol followed by the incubation with extraction buffer to extract keratin. Extraction buffer composed of urea, thiourea, betamercaptoethanol and tris–HCl. The obtained protein extract was dialyzed and lyophilized to obtain the protein powder which was further utilized to obtain bioplastic film by solution casting with various plasticizers. See text for details.

The keratin from delipidized human hair samples was isolated by Shindai Method^33^ and quantified by Bradford method^34^. Protein concentration after 3 days of extraction at 50 °C was around 2.9 mg/ml and that after 6 days of extraction period was about 9.8 mg/ml. The extracted protein solution was dialyzed and lyophilized to obtain the keratin protein powder. Lyophilization of protein solution gave a yield of 28 ± 2%. The lyophilized protein was subjected to SDS PAGE analysis to determine the molecular weight composition of the isolated protein sample. The isolated protein gave two bands, first at 35–48 kDa representing the microfibril component and second at 10–20 kDa corresponding to matrix proteins. The isolated protein was then utilized to prepare the bioplastic film by solution casting in silicon moulds and final curing (Fig. 1). Various plasticizers viz. glycerol, ethanediol, diethylene glycol and triethylene glycol were used to form the bioplastic film and were characterized by FESEM, FTIR and XRD techniques.

### Morphological characteristics of the keratin bioplastic

**Figure 2:**
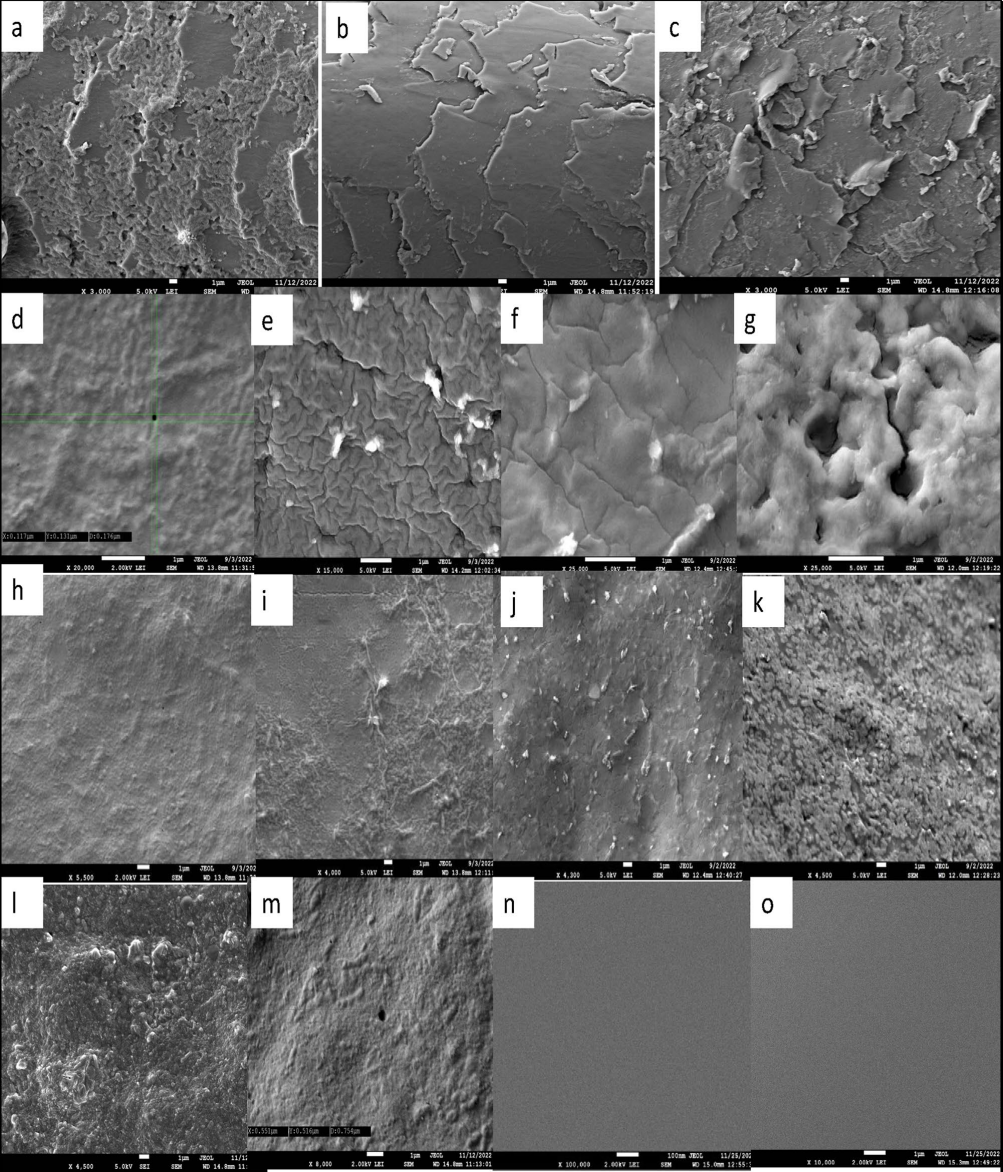
Surface morphology of bioplastic films. (**a–c**) FESEM images of virgin hair, delipidized hair and hair after extraction respectively. (**d–g**) FESEM images of G100, G500, G1000 and G1500 at ×20,000–×25,000 respectively-G100 shows a pit with size 0.1 µm, G500 shows wrinkled appearance, G1000 displayed tiled appearance and G1500 showed larger pits of about 1.4 µm. (**h–k**) FESEM images of G100, G500, G1000 and G1500 at ×4000 to ×5000 respectively. (**l,m**) FESEM images of ED100 at ×4500 and ×8000 respectively. (**n,o**) FESEM images of DEG100 and TEG100 respectively.

Field emission scanning electron microscopy (FESEM) was utilized to analyze the morphological characteristics of the bioplastic films formed with various plasticizers. Also the virgin hair delipidized hair and hair fragments after protein extraction were analyzed to access the changes during the extraction procedures. Imaging of virgin hair at X3000 showed the presence of lipid depositions between the cuticle scales (Fig. 2a). The analysis of hair fragments after lipid removal showed the absence of lipid depositions and depicted smoother appearance (Fig. 2b), that after extraction showed the distorted morphology of the hair scales and scales were broken and disrupted as a results of the action of reducing and denaturing agents from the extraction buffer (Fig. 2c).

In order to access the suitability of glycerol as plasticizer and optimize the concentration required for the formation of stable bioplastic film varied amount of 1% glycerol were first utilized for solution casting and film formation. Films with 100, 500, 1000 and 1500 µl of 1% glycerol with 10 mg of keratin powder were formed and analyzed. FESEM images of films at about ×20,000 of G100, G500, G1000 and G1500 are shown in Fig. 2d–g respectively and that at about ×4000 are shown in Fig. 2h–k.

The surface of G100 films at lower magnifications of ×300 was observed to be continuous with certain ups and downs (Supplementary Fig F1a) with these lower magnifications no holes or pits were visible. Upon further increasing the magnification at ×5000 (Supplementary Fig. F1a) just one or two pits were seen with an approximate size of 0.1 µm recorded at ×20,000 (Fig. 2d), 2 kV. These pits were not holes or tunnels and were formed as a result of discontinuous intermolecular interactions between the keratin molecules in the upper layers only and the layers underneath appeared to be continuous.

The analysis of G500 films at ×800 depicted smooth and continuous surface (Supplementary Fig. F1b). At ×4000, the morphology of the film was found to be irregular with certain bumps and bulges but no pits or holes were seen (Fig. 2i). At higher magnification (×15,000), Fig. 2e, the film displayed wrinkled appearance nevertheless no holes or pits were visible. These films were found to be sensitive to higher voltages and the specimen showed ruptures at further higher magnifications (Supplementary Fig. F1b).

Further increasing the concentration of glycerol deteriorated the surface morphology of the bioplastic films, hence G1000 and G1500 displayed non-uniform surface even at ×400 (Supplementary Fig. F1c). Imaging at ×4000 revealed continuous surface in the films along with certain filamentous depositions (Fig. 2j). Observations at ×12,000 (Supplementary Fig. F1c) and ×25,000 (Fig. 2f) displayed tiled appearance with no holes or pits. G1000 films were found insensitive to operating voltages of up to 5.0 kV.

The surface structures of G1500 films appeared to be extremely discontinuous at ×23,000 (Fig. 2g), with the uppermost layer being non uniform and the inner layers seems to be continuous. At lower magnifications at around ×8000, these films showed granular appearance (Supplementary Fig. F1d). Larger pits of about 1.8 µm were observed (Supplementary Fig. F1d) and the discontinuous and granular appearance of these films could be attributed to the accumulation of excess glycerol.

From the comparative analysis of all these glycerol films, it was concluded that the G100 films displayed the best morphological characteristics and hence same concentration of other plasticizers were taken up for further studies. In the next set of trials films were formed with 100 µl 1% solution of ED, DEG and TEG to yield ED100, DEG100 AND TEG100 films with the same protocol as applied for glycerol films.

ED100 films displayed distinct surface morphology as observed in SEI and LEI mode. At lower magnifications, ED100 films showed similar surface morphology as G100 films (Supplementary Fig. F1e). Pit of size 0.75 µm was recorded at ×8000 (Fig. 2l, m) and at the similar magnifications, under SEI imaging the films appeared to have wrinkled appearance (Supplementary Fig. F1f). At further higher magnification of around ×20,000, the surface appeared to be wrinkled in LEI mode (Supplementary Fig. F1g).

As seen under varied magnifications, the surface structures of the DEG100 and TEG100 films were found to be smooth (Fig. 2n–o). No pits, holes, bulges or wrinkles were visible in DEG100 and TEG100 films at around ×100,000 and above magnifications. Overall comparison in all the films suggested that DEG100 and TEG100 films have the most uniform surface morphology followed by G100 and ED100.

### XRD diffractogram depicted the crystalline characteristics of the keratin films

**Figure 3:**
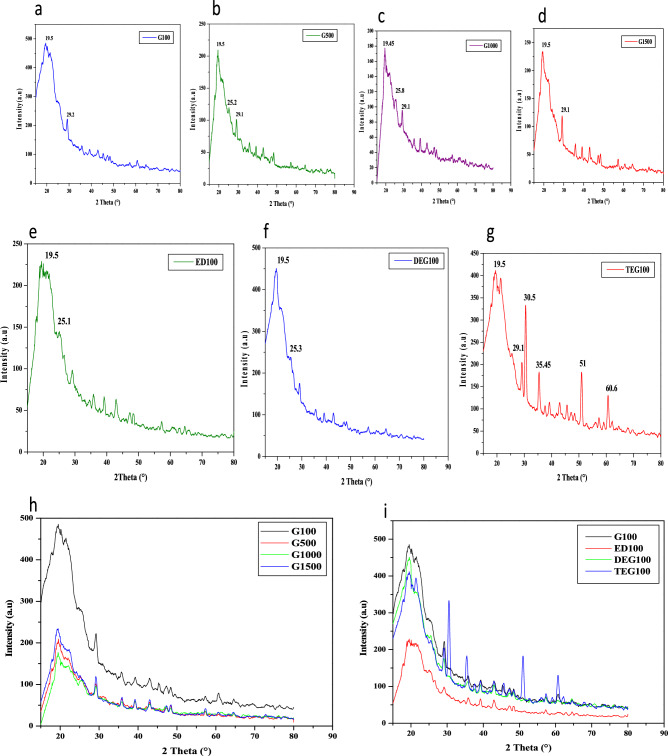
XRD diffractogram of bioplastic films. (**a–d**) Diffractogram of G100, G500, G1000 and G15000 films. (**e–g**) Diffractogram of D100, DEG100 and TEG100 films. (**h**) Comparative diffractogram of glycerol films, G100 films showing the most intense peak at 19.5°. (**i**) Comparative diffractogram of G100, ED100, DEG100 and TEG100 films.

The X-ray diffractograms were obtained for all the keratin films in the 2θ range of 15° to 85° (Fig. 3a–d). Firstly the XRD analysis was conducted for all the glycerol films viz. G100, G500, G1000 and G1500. In all these films, the characteristic peak of keratin was observed at around 19.5°. In G100 and G500 and G1500 films the peak was observed at 19.5° and in G1000 films it was observed at 19.45°. In addition to this characteristic peak, additional less intense peaks were also observed at different 2θ values. In G100, the minor peak was observed at 29.2° and in other glycerol films it was observed at 29.1°. As intense peak was observed in all the glycerol films, at around 2θ values of 19.5°, this could be attributed to the crystalline behavior of these films. By applying the Bragg’s law the inter atomic spacing or d value for these films were found to be 0.455 nm for G100, G500 and G1500 films and 0.457 nm for G100 films respectively. Overall d value of glycerol films was found to be significantly similar with a difference in value of about 0.002 nm.

The comparative diffractogram for all the glycerol films with different concentration of plasticizer (Fig. 3h) shows a clear distinction between G100 and other films. Most intense peak was observed in G100 films at 485.6 a.u followed by G1500, G500 and G1000 at 234.51, 209.59 and 177.51 a.u respectively. The highest peak intensity in G100 films could be attributed to their most crystalline nature among all the glycerol plasticized keratin films.

In the later stages of study, the films formed with other plasticizers were also analyzed and X ray diffractogram for these films were recorded in the range of 15° to 85° (Fig. 3e–g). Use of ED, DEG and TEG as plasticizers in human hair keratin film is being reported for the first time in present study. Depicting the similar behavior as the glycerol plasticized keratin films, ED100, DEG100 and TEG100 films showed the characteristic peak for keratin at 19.5° as well. The minor peaks were recorded at 25.1° and 25.3° in ED100 and DEG100 films. In TEG100 films, additional sharp peaks were recorded at 30.5°, 35.45°, 51° and 60.6° that could be attributed to increased crystalline behavior in these films as a result of additional interactions between TEG and the amino acid side chains of the keratin molecules.

The d spacing value in ED100, DEG100 and TEG100 films was found to be 0.455 nm which is same as that of glycerol films. The comparative analysis of all the films depicts the most intense peak in G100 films, followed by DEG100, TEG100 and ED100 being the least (Fig. 3i). Glycerol films displayed the most crystalline nature among all the films.

### FTIR showed the changes in amide peaks with different plasticizers

FTIR analysis was utilized to analyze the functional groups and their behavior in the keratin films formed with different plasticizers. Lyophilized keratin powder was also analyzed by FTIR studies and the absorption and transmittance plots of the same were also compared with that of the films to analyze the change in bond stretching and amide peaks while the film formation. The peaks corresponding to Amide A, Amide I, Amide II and Amide III were located in all the samples and changes in them were analyzed.

**Figure 4:**
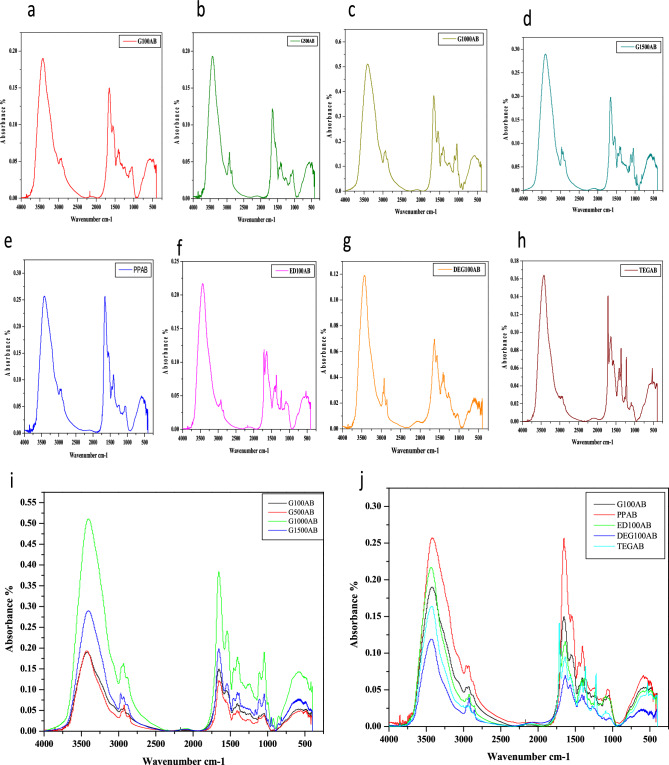
FTIR absorbance spectra. (**a–d**) Infrared absorbance spectra of G100, G500, G1000 and G1500 respectively. (**e**) IR absorbance spectra of lyophilized keratin. (**f–h**) IR absorbance spectra of ED100, DEG100 and TEG100. (**i**) Comparative absorbance spectra of glycerol films at various concentrations. (**j**) Comparative absorbance spectra of lyophilized keratin, G100, ED100, DEG100 and TEG100 films.

The absorbance spectra of lyophilized keratin are given in Fig. 4e and that of G100, G500, G1000 and G1500 are shown in Fig. 4a–d respectively. ED100, DEG100 and TEG100 absorbance spectra are given in Fig. 4f–h respectively. The comparative absorption spectrum of all glycerol films is given in Fig. 4i and that of films with all the plasticizers and lyophilized keratin is shown in Fig. 4j.The analysis of comparative spectra of glycerol films suggests that these films show the absorption peaks at similar wavenumbers. Most intense peak was observed in G1000 films that could be attributed to their increased moisture content relative to films with lower concentrations of glycerol.

**Figure 5:**
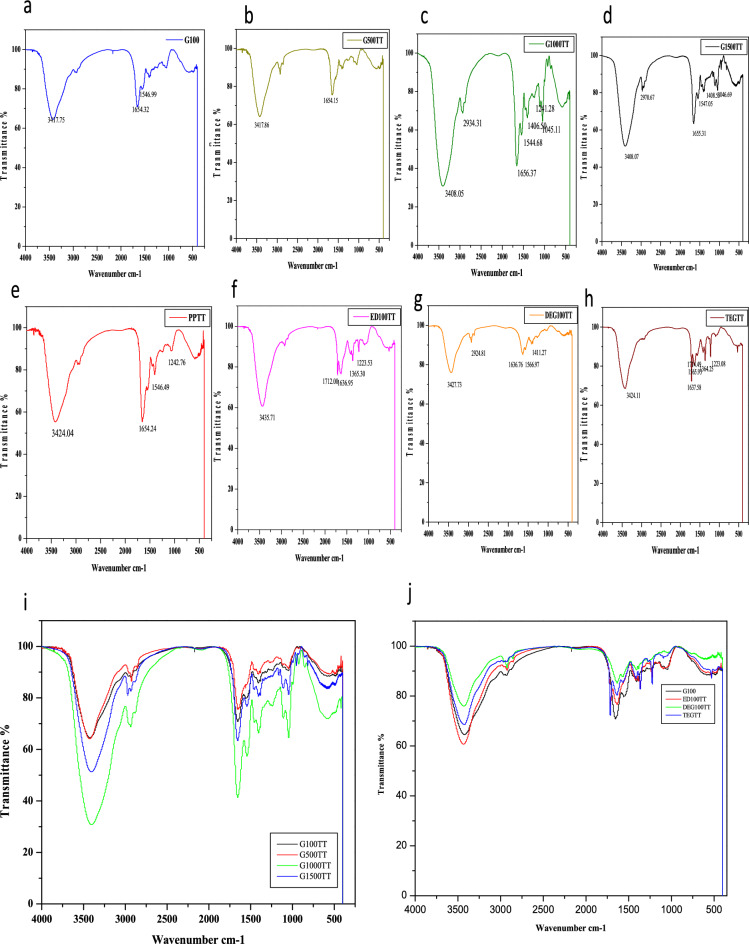
FTIR transmittance spectra. (**a–d**) Infrared transmittance spectra of G100, G500, G1000 and G1500 respectively. (**e**) IR transmittance spectra of lyophilized keratin. (**f–h**) IR transmittance spectra of ED100, DEG100 and TEG100. (**i**) Comparative transmittance spectra of glycerol films at various concentrations. (**j**) Comparative transmittance spectra of G100, ED100, DEG100 and TEG100 films.

Similarly while observing the other films comparative absorption spectra with that of lyophilized protein, most intense peak was observed in lyophilized keratin and the least was observed in DEG100 films that could be again be due to difference in their moisture contents. Transmittance plots of G100, G500, G1000 and G1500 films are given in Fig. 5a–d and that of lyophilized keratin, ED100, DEG100, TEG100 films are given in Fig. 5e–h. Transmission peaks observed in all the films are given in Table 1.

**Table 1:** IR Transmission peaks for keratin bioplastic films.

Sample	Amide I	Amide II	Amide III	Amide A	Others
Lyophilized keratin	1654.24	1546.49	1242.76	3424.04	–
G100	1654.32	1546.99	–	3417.75	–
G500	1654.15	1546.69	–	3417.86	–
G1000	1656.37	1544.68	1241.28	3408.05	2934.31 (C–H stretch, methylene), 1406.50 (ammonium ion), 1045.11 (phosphate ion)
G1500	1655.31	1547.05	–	3408.07	1046.69 (phosphate ion), 1408.55 (ammonium ion), 2970.67 (CH stretch, methyl)
ED100	1636.95	–	1223.53	3435.71	1365.3 (dimethyl/trimethyl), 1712.00 (carboxylic acid, ketone)
DEG100	1636.76	1566.97	–	3427.73	1411.27 (methyl: C–H bend), 2924.81 (methylene; C–H stretch),
TEG100	1637.58	1565.93	1223.08	3424.11	1364.25 (di-methy), 1714.49 (carboxyl)

In lyophilized protein the Amide A broad peak was seen at 3424.04 cm^−1^ with peak intensity 55.35 a.u. Amide I, Amide II and Amide III peaks were recorded at 1654.24, 1546.49, 1242.76 cm^−1^ respectively. G100 films showed two characteristic peaks, one broad peak for Amide A at 3417.75 cm^−1^ and for Amide I at 1654.32 cm^−1^. The minor peak for Amide II was recorded at 1546.99 cm^−1^ but that for Amide III was not observed. Transmission peaks observed in G500 are at 3417.86, 1654.15 and 1546.69 cm^−1^ for Amide A, Amide I and Amide II respectively. The positions for amide peaks were found to similar in both G100 and G500 films.

Multiple peaks were observed in G1000 and G1500 films in addition to the characteristics peak of keratin. The characteristic peaks for Amide A, Amide I, Amide II and Amide III were found to be at 3408.05, 1656.37, 1544.68 and 1241.28 cm^−1^ in G1000 film. Along with these, the peaks at 1045.11 cm^−1^ and 1406.50 cm^−1^ could be attributed to phosphate and ammonium ion contributions from the amino acid side chains. Similarly the peak at 2934.31 cm^−1^ represented the increased C–H stretch at methylene in side chains in protein chain^35–37^.

The characteristics peaks observed in ED100 films transmittance plots were at 3435.71, 1636.95 and 1223.53 cm^−1^ for Amide A, Amide I and Amide III respectively. Additional peak observed at 1712.00 cm^−1^ could be attributed to carboxyl group from amino acid side chain. The characteristic peaks observed in the transmittance plots of DEG100 films were at 3427.73, 1636.76 and 1566.97 cm^−1^ for Amide A, Amide I and Amide II respectively. Peaks at 1411.27 cm^−1^ and 2924.81 cm^−1^ correspond to C–H stretching in the side chains along the polypeptide chain. TEG100 transmission peaks for Amide A, Amide I, Amide II and Amide III were recorded at 3424.11, 1637.58, 1565.93 and 1223.08 cm^−1^ respectively along with additional peaks present at 1364.25 and 1714.49 cm^−1^ which may be due to additional interactions among proteins side chains and plasticizer molecules. Comparative transmittance plots of all the glycerol films and that of other plasticizers are given in Fig. 5i, j respectively.

The position of Amide I peak suggests the global secondary structure present in the protein. The interpretation of Amide I peaks in all the films and the respective predicted secondary structures are given in Table 2. It was recorded that, the native alpha helix conformations were somewhat preserved in glycerol films although there might be some distortions. Whereas it was observed that the Amide I peak for ED100, DEG100 and TEG100 falls in the region of beta sheets suggesting a conformational change in the native protein structure during the film formation due to interactions with the plasticizers. This analysis suggested that the molecular characteristics of the films prepared with glycerol are different from that prepared with other plasticizers.

**Table 2:** Predicted secondary structures of keratin in bio-plastic films.

Film	Amide I peak (cm^−1^)	Global secondary structure
G100	1654.32	Alpha helix
G500	1654.15	Alpha helix
G1000	1656.37	Alpha helix
G1500	1655.31	Alpha helix
ED100	1636.95	Beta sheet
DEG100	1636.76	Beta sheet
TEG100	1637.58	Beta sheet

### Dimensions and water solubility of bioplastic film

The film prepared were of the thickness 190–220 µm with the area of about 4.54 ± 0.2 cm^2^. Water uptake by G100, ED100, DEG100 and TEG100 films was recorded to be 4.8, 6.2, 4.9 and 6.3% respectively. Maximum water uptake was recorded by TEG100 films and least by G100 films.

### Biodegradation by *A. oryzae*

**Figure 6:**
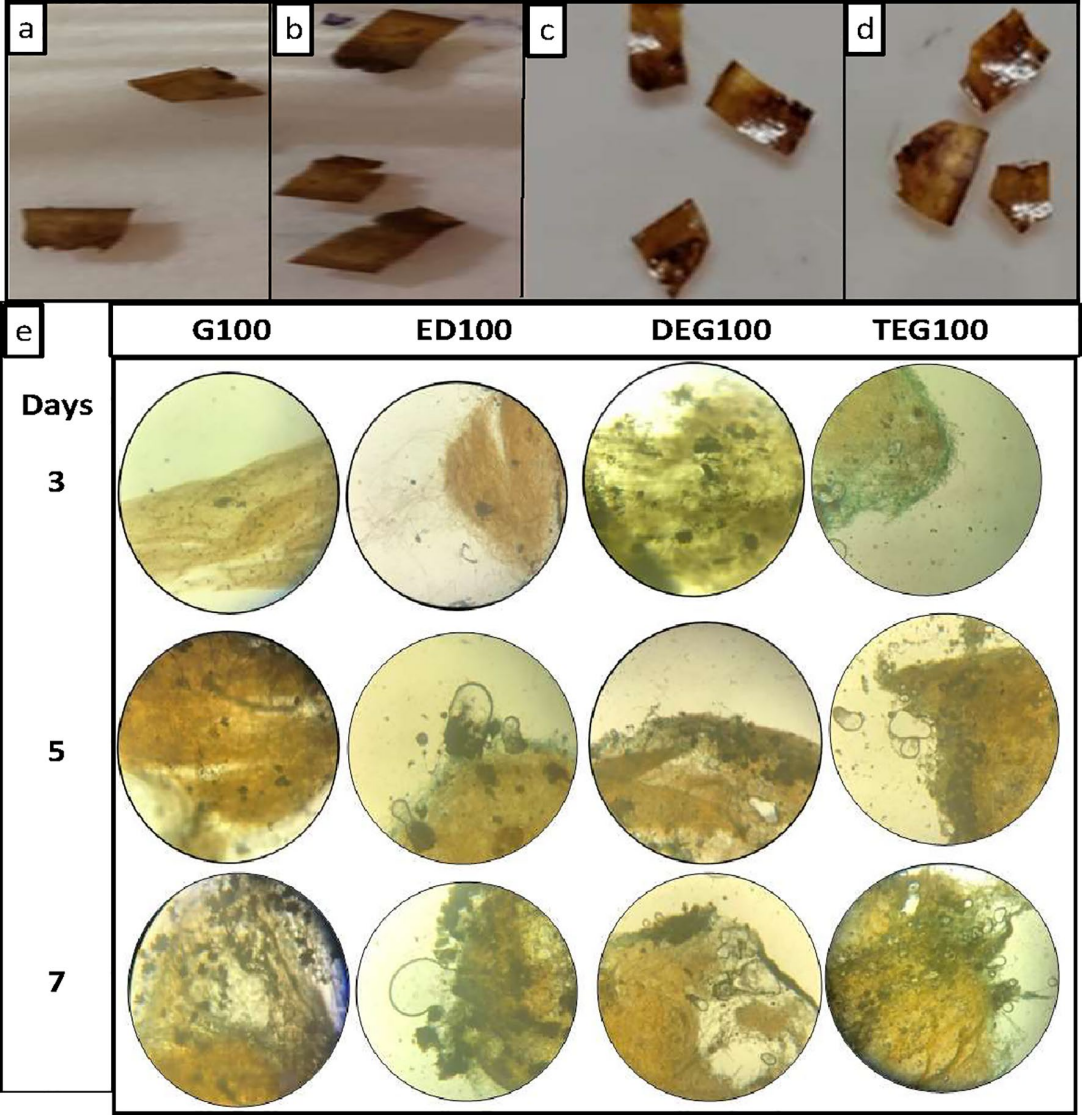
Bioplastic films. (**a–d**) G100, ED100, DEG100 and TEG100 respectively. (**e**) Microscopic images of film degradation by *A. oryzae* after different time intervals (3, 5 and 7 days).

Degradation of G100, ED100, DEG100 and TEG100 films (Fig. 6a–d) were studied by using keratinophilic isolate of *A. oryzae*. The films when incubated with *A.oryzae* depicted degradation to about 80% after 7 days. The fungus was observed to maintain good association on the surface of the film and was able to degrade it on the surface as well as penetrated and degraded the internal portions (Fig. 6e).

## Discussion

The exponential increase in the demands of synthetic plastics in each and every sector of human society coupled with unconscious and unjust disposal in majority of scenarios have made it indispensible to produce more environmental friendly plastics that are biodegradable and are derived from renewable resources. Animal proteins like Collagen, Milk proteins, Myofibrillar proteins, Keratin and Gelatin have been utilized for the production of bioplastics^38–43^. Some of the plant proteins utilized for the same purpose includes gluten, soy protein and zein^44–48^. Also, others include bio-waste and co- products like co-products from starch, bio-ethanol and seed oil, microalgae from sewage and agricultural farming waste^49^.

With this aim, in present study we explored the use of keratin from human hair waste to analyze its potential as a candidate to produce bioplastic films. Keratin from human hair was utilized to form the bioplastic films by solution casting in silicon moulds. Effect of different plasticizers like glycerol, ED, DEG and TEG were analyzed. Glycerol has been shown to be useful in the formation of keratin film from human hair and poultry waste. Glycerol as plasticizer act by reducing the intermolecular interactions between the protein chains and allows free movement in them. Glycerol also improves the resistance to breaking and cracking in the bioplastic film and improves the physical characteristics of films.

We have demonstrated the effect of various concentrations of glycerol on the properties of the film. By using 1% solution of glycerol, it was found that the G100 films depicted the most uniform surface morphology and by increasing the concentration of the plasticizers the time for drying of the film increased from about 48 h to almost 15–20 days, moreover the film surface showed excessive depositions and granular appearance. The increase in the drying time of the films with increased concentrations of glycerol could be attributed to the fact that excess glycerol makes the bioplastic material more susceptible to absorbing water and prolonged the water removal from the film surface. Moreover, the changed surface morphology with excess depositions emphasizes the importance of the right amount of glycerol to be added in order to achieve desirable film surface properties. It could also be noted that excessive glycerol can lead to weaker intermolecular interactions, resulting in films that are more susceptible to breaking. The data for human hair keratin film with varied concentration of glycerol is unavailable alas; studies are available showing good morphology under SEM with no holes in chicken feather keratin films with 2% glycerol^21^. Similarly chicken feather keratin film and microcrystalline cellulose with no holes or cavities in the SEM studies have been reported^50^.

Keratin films with porous structure and pore size 0.1–0.2 µm prepared in Teflon rings and siliconized base, films with 1% glycerol in petri plates having a non-homogenous morphology have been reportedpreviously^30,51^. Therefore our adopted method of using silicon moulds to prepare films was found to be better than previously reported methods as it yielded films with continuous and homogenous morphology with fewer and smaller pits.

ED100 films also showed similar surface morphology as G100 films with minor pits of average size 0.75 µm. DEG100 and TEG100 films also possessed smooth surface with no pits or holes. The better surface characteristics with DEG100 and TEG100 films could be due to enhanced amino acid chain mobility leading to improved distribution of the proteins during processing. Also, the surface defects in the protein films arise because of internal stresses^52^, DEG and TEG tends to reduce this stress owing to better chain alignment and reducing molecular entanglements. As a result, the surface of the film becomes smoother and more uniform with these plasticizers in comparison to ED and glycerol.

Noting the crystalline nature of these films as recorded by XRD analysis, the d-spacing value was 0.455  ± 0.002 nm irrespective of the plasticizer used and G100 and DEG100 films showed the most crystalline behavior and ED100 possessed least crystalline nature as compared to all the other films. Nevertheless, the characteristic peak of keratin was observed at around 19.5° in all the films. Moreover, additional peaks were recorded in the TEG100 films, owing to the increased interactions between the polypeptide side chain and the plasticizer molecules. Lattice spacing of lyophilized keratin extracted by similar method is also 4.5 Å^42^. According to previous reports, human hair keratin film with 1% glycerol possessed diffraction peak at 19.9°, chicken feather keratin film at 19°, 23° and 41° and keratin cellulose films at 19°, 32°, 41°, 45°and 66° ^50,51^.

The FTIR analysis of the films showed presence of various Amide peaks in the transmittance plots. Moreover, the peak intensity for Amide A and amide I reduced with increased concentration of the glycerol and the highest peak intensity were observed in DEG100 films followed by TEG100 films. Thissuggested the presence of increased H bonded peptides in TEG100 and DEG100 films. The peak of Amide A for human hair keratin isolated by urea have been reported at 3276 cm^−1^ and Amide I, Amide II and Amide III at 1640, 1517 and 1234 cm^−1^ respectively^27^. Scaffolds prepared with keratin-PVA with alginate dialdehyde as cross-linking reagent recorded peaks at 3422.06, 1642.84, 1460.81 cm^−1^ ^53^. Similar peaks in IR spectrum are shown by the regenerated keratin and films prepared from chicken feather keratin^21,30,50^. All the keratin films showed water uptake in water solubility test which was recorded to be highest in TEG100 films displaying its hydrophilic nature, owing to increased probability of forming hydrogen bonding. Nevertheless, all the films showed partial solubility in water that could be of importance in specific applications.

In the present research, degradation by *A. oryzae* of human hair keratin films is being studied for the first time, nevertheless, the growth of *Trichophyton rubrum *on the human hair keratin film have shown that the fungus could grow on the surface and also penetrated these films^51^.

In biodegradation studies using keratinophilic fungi (*A. oryzae*), the keratin films showed significant level of degradation of about 80% after seven days of incubation. The keratinases enzymes produced by the fungi, helped it in the penetration and further degradation of the keratin films^54^. The biodegradability of these films is of significant importance as far as their future applications in packaging or other industry is concerned.

## Conclusion

The present study involved the preparation and characterization of the keratin films or ‘kertics’ from human hair waste and exploring the potential of novel plasticizers. The addition of DEG and TEG as plasticizer in the keratin films improved their morphological properties. The characteristics and behavior of these films could be modulated by employing different plasticizers and by modulating the quantity of these plasticizers for specific applications. Moreover the keratin films were found to be biodegradable that makes them suitable to be utilized as a sustainable material in future applications as an alternate to conventional plastics. Kertics could be utilized for packaging application and also for the generation of materials suitable for various biomedical uses. The outcomes of the present study shall contribute in the better understanding of the human hair based keratin films and help in exploring more opportunities for their use. The mechanical properties of kertics, such as tensile strength, elongation at break and E-modulus could be analyzed in future studies to assess the strength and suitability of these films for various applications.

## Methods

### Collection of hair samples

Hair samples were collected from volunteers with prior informed consent from the salons in Agra, Kamla Nagar and Dayalbagh region. The collected hair samples were cleaned by removing the coarse dust particles by hand and thereafter stored in sterile bags for further analysis. All experiments were performed in accordance with relevant guidelines and regulations. All the methods adopted in the present study were reviewed and approved by Dayalbagh Educational Institute, Dayalbagh, Agra, India.

### Methods for film formation

#### Hair delipidization

The collected hair samples was washed in doubled distilled water followed by mild soap and dried in oven overnight at 40 °C. The cleaned and dried hair samples were then soaked in chloroform and methanol in the ratio 2:1 for 48 h at room temperature in order to remove the lipids. After delipidization, the solvents were drained off and the hair samples were again washed twice with chloroform and methanol solution to completely remove the sticking lipid globules if any, thereafter the hair were again washed with ddH_2_O, dried and used for protein extraction.

#### Extraction of hair keratin

The delipidized hair samples were cut into small fragments (1–2 mm) with the help of scissors. 20 g hair samples were then incubated with extraction buffer composed of trisHCl (25 mM, pH 8.5), thiourea (2.6 M), urea (5 M) and mercaptoethanol (5%) at 50 °C, 75 rpm^14^. The extraction was done in shaker incubator for 3 to 6 days. The protein extract was retrieved by sieving through medical gauge, filtration and centrifugation at 4500 rpm for 20 min.

#### Dialysis of protein extract

The obtained protein extract was to completely remove the extraction buffer components. The dialysis was carried out with the Spectra Por S/P Dialysis Membrane, with MWCO 6000–8000 Da for 3 days against ddH_2_0. The water was changed after every 12 h of interval. The dialyzed protein extract was stored at −90 °C and thawed before further processing.

#### Quantification of protein

Quantification of protein in the dialyzed protein extract was carried out by Bradford assay using Himedia HiGenoMB Bradford Reagent (ML106) and HiMedia bovine serum albumin (BSA) (MB083) as a standard.

#### Lyophilization of dialyzed extract

The dialyzed protein extract was freeze dried or lyophilized by freezing the extract and reducing the surrounding pressure to directly sublime the water from solid to gas phase. The lyophilization of the protein samples was done by Lyophilizer-FD 5.

#### SDS-PAGE analysis

For the analysis of molecular weight composition of the protein extract sodium dodecyl sulfate polyacrylamide gel electrophoresis (SDS-PAGE) was utilized. The protein samples were in the gel (10 × 10 cm). Separation was carried out on Analytical single moulded vertical electrophoretic system using standard protein ladder of 245 to 11 kDa. Coomassie brilliant blue dye was used for staining and the gel was thereafter de-stained using de-staining solution comprising of methanol, acetic acid and water in the ratio 5:1:4. Protein bands were then visualized.

#### Solution casting for film formation

10 mg of keratin powder was redissolved in ddH_2_O and was then mixed with 100, 500, 1000 and 1500 µl of 1% glycerol solution in first set of trials. The resulting mixtures of glycerol and keratin solution was conditioned for 4 h at 50 °C under shaking conditions and then casted in silicon moulds. The films were kept for drying at 50 °C using dry heat. In the next set of trials 100 µl of 1% solutions of ethanediol (ED), diethylene glycol (DEG) and tri-ethylene glycol (TEG) were used to form the protein bioplastic films, rest of the procedures were kept same.

#### Curing of the bioplastic films

The preliminary films obtained after solution casting were then subjected to curing temperature of 110 °C for 3 h. Post curing, the films were then subjected to reduced temperatures (approximately 4 °C). The resultant hard films were then taken up for characterization studies.

### Methods for characterization

#### Morphological characterization by FESEM

Field Emission Scanning Electron Microscope was utilized to analyze the surface morphology of the hair fragments at various stages of the studies. Morphological characteristics of the bioplastic films obtained with different plasticizers were also revealed by using FESEM analysis. Before analysis the samples were platinum coated with the help of JEOL, JEC-3000FC auto fine platinum coater. Analysis was made FESEM-JEOL 7610F PLUS at magnifications ranging from ×300 to ×3000 in both lower electron imaging (LEI) and secondary electron imaging (SEI) mode as suitable depending on the sample in study.

#### Characterization by XRD

X-ray diffraction is utilized in order to analyze the crystalline structure of the polymeric substances. The analysis of Bragg peaks scattered to wide angles is made during this study. The analysis of Bragg peaks scattering caused by nanometer and sub-nanometer sized structures is done. The percentage crystallinity of the sample could be determined using this technique. The scattering intensity is plotted as a function of 2θ angle and the analysis of the same is made. This is a non destructive method of characterization. In a crystalline solid, the distance between the imaginary planes in which the atoms are regularly spaced is known as d-spacing. This d-spacing is specific to every material. The structure of keratin bioplastic membranes in the present study was analyzed by D8 ADVANCE X-Ray Diffractometer.

#### Characterization by FTIR

Fourier-Transform Infrared Spectroscopy provides the information about infrared spectrum of absorption or emission of a solid, liquid or gas samples. The information of these patterns could be exploited to identify the functional groups present in the samples. This may also help to analyze the changes, addition or removal of functional groups as a result of the processing of protein in the formation of bioplastic films. Films prepared with glycerol, ED, DEG and TEG were analyzed by FTIR NICOLET-IS-50 over a range of 400 to 4000 cm^−1^. Sample preparation was done by mixing with KBr in ratio 1:100 (w/w) and grinding vigorously thereafter making pellet by using a press for analysis.

#### Dimensions and water solubility of bioplastic films

Film thickness was measured with the help of micrometer. Measurement of the radius was done by ruler and used for the calculating the area of the bioplastic samples.

#### Biodegradation test by *A. oryzae*

The isolation of keratinophilic fungi was done from soil by To-ka-Va hair bating technique^55^. Molecular identification of the isolate was done by ITSrRNA gene sequencing, conducted at NCIM Pune, India. For the degradation studies, the bioplastic substrates were incubated with fungal cultures and 10 ml basal solution in petri plates. Observations were made at the interval of 3, 5 and 7 days under optical microscope.

### Supplementary Information

Supplementary Figure 1.

## Data Availability

The datasets used and/or analysed during the current study available from the corresponding author on reasonable request.
