# Supplementary material for: Production and characterization of human hair keratin bioplastic films with novel plasticizers
Source: Sci Rep. 2024 Jan 12;14:1186. doi: 10.1038/s41598-023-44905-x (PMC10786936; doi:10.1038/s41598-023-44905-x)
Supplement: Supplementary file 1 — Supplementary Figure 1. [file 41598_2023_44905_MOESM1_ESM.pdf]

Production and characterization of human hair keratin bioplastic films with novel plasticizers

Anand Shubha, *Gupta Sharmita, Lakhani Anita

Dayalbagh Educational Institute, Dayalbagh, Agra-282005, U.P., India

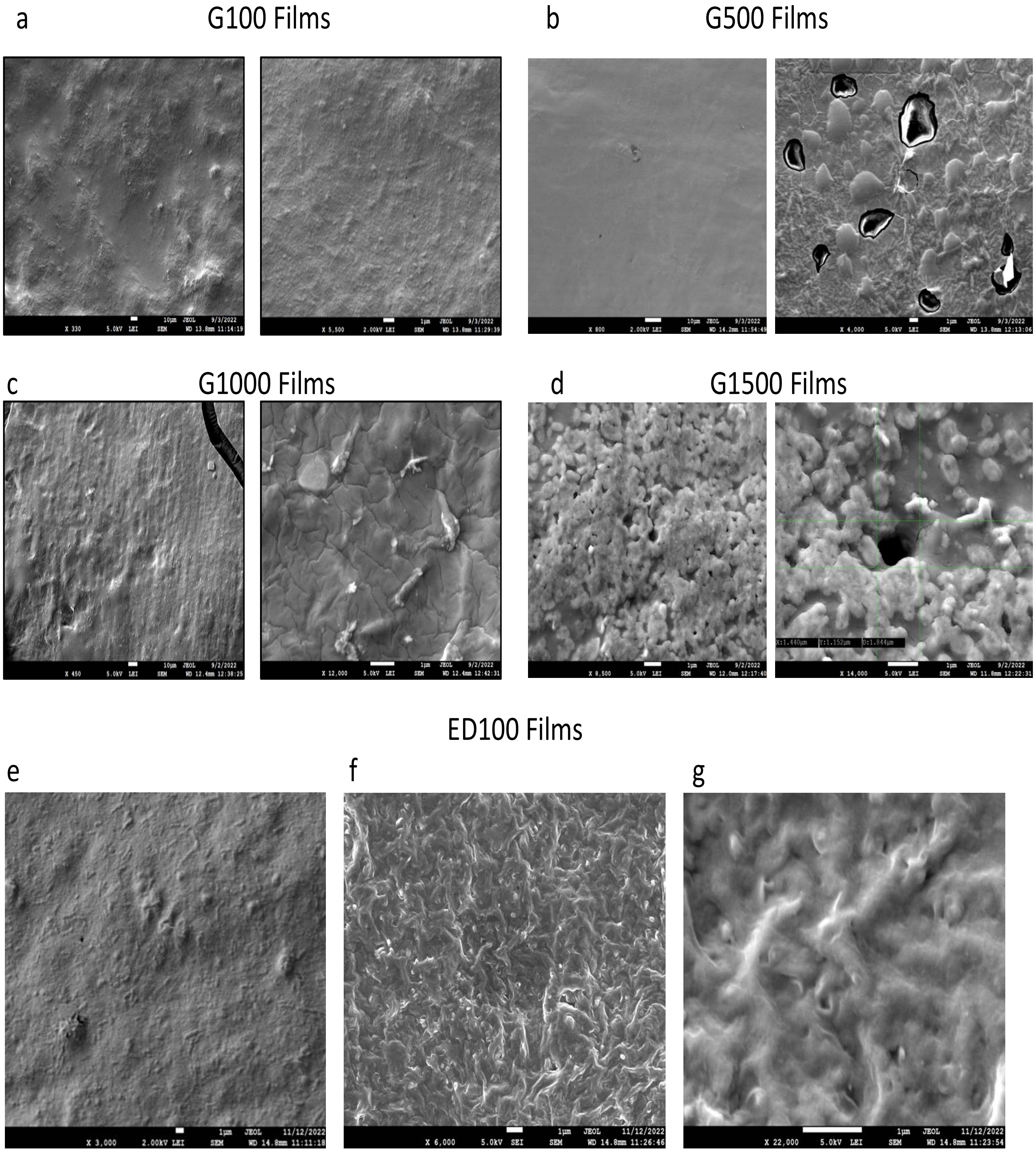

Supplementary Fig1 : FESEM images of keratin films. a, G100 films at X300 and X5000. b, G500 films at X800 and X4,000. c, G1000 films at X400 and X12000. d, G1500 at X8000 and X14000. e, ED100 films at X3000 in LEI mode, X6000 in SEI mode and X22000 in LEI mode.

Supplementary Fig F1: FESEM images of keratin films. a, G100 films at X300 and X5000. b, G500 films at X800 and X4,000. c, G1000 films at X400 and X12000. d, G1500 at X8000 and X14000. e, ED100 films at X3000 in LEI mode, X6000 in SEI mode and X22000 in LEI mode.
